# Comparative Analysis of Molecular RFLP and SNP Markers in Assessing and Understanding the Genetic Diversity of Various Chicken Breeds

**DOI:** 10.3390/genes13101876

**Published:** 2022-10-16

**Authors:** Natalia V. Dementieva, Yuri S. Shcherbakov, Valentina I. Tyshchenko, Valeriy P. Terletsky, Anatoly B. Vakhrameev, Olga A. Nikolaeva, Anna E. Ryabova, Anastasiia I. Azovtseva, Olga V. Mitrofanova, Grigoriy K. Peglivanyan, Natalia R. Reinbah, Darren K. Griffin, Michael N. Romanov

**Affiliations:** 1 Russian Research Institute of Farm Animal Genetics and Breeding—Branch of the L.K. Ernst Federal Research Centre for Animal Husbandry, Pushkin, 196601 St. Petersburg, Russia; 2 Pushkin Leningrad State University, Pushkin, 196605 St. Petersburg, Russia; 3 School of Biosciences, University of Kent, Canterbury CT2 7NJ, UK

**Keywords:** chicken breeds, divergent selection, genetic diversity, RFLPs, DNA fingerprinting, genome-wide SNP genotyping, phylogeny, gene pool

## Abstract

Monitoring the genetic diversity of small populations is important with respect to conserving rare and valuable chicken breeds, as well as discovery and innovation in germplasm research and application. Restriction fragment length polymorphisms (RFLPs), the molecular markers that underlie multilocus DNA fingerprinting (MLDF), have historically been employed for this purpose, but over the past two decades, there has been an irreversible shift toward high-throughput single-nucleotide polymorphisms (SNPs). In this study, we conducted a comparative analysis of archived MLDF results and new data from whole-genome SNP genotyping (SNPg) among 18 divergently selected breeds representing a large sample of the world gene pool. As a result, we obtained data that fit the general concept of the phylogenetic distribution of the studied breeds and compared them with RFLP and SNP markers. RFLPs were found to be useful markers for retrospective assessment of changes in the genetic architecture and variability underlying the phenotypic variation in chicken populations, especially when samples from previous generations used for MLDF are unavailable for SNPg. These results can facilitate further research necessary to assess the possibility of extrapolating previous MLDF results to study the long-term dynamics of genetic diversity in various small chicken germplasm populations over time. In general, the whole-genome characterization of populations and breeds by multiple SNP loci will further form the basis for the development and implementation of genomic selection with the aim of effective use of the genetic potential of the domestic gene pool in the poultry industry.

## 1. Introduction

Currently, poultry farming is the most efficient branch of animal husbandry, and domestic chicken (*Gallus gallus*) is the most common type of poultry [1]. Many years of divergent selection have resulted in a considerable variety of chicken breeds and populations that differ in terms of phenotypic variation, utility type, performance, and unique genetic architecture (e.g., [2,3,4,5]). The goals of gene pool conservation are not only to preserve genetic diversity but also to use individual breeds as sources of valuable genes, variants, and their combinations to develop new lines/breeds and respond to market volatility [6,7,8,9]. An analysis of the genetic variability of breeds is a necessary step for the subsequent successful prediction of the breeding effect (e.g., genomic selection) and to understand the biological mechanism of adaptive and other features of chicken breeds (e.g., [10]).

Modern methods of molecular genetic analysis are rapidly developing and improving (e.g., [11,12,13]). On the other hand, previous studies conducted using genome-wide methods, such as multilocus DNA fingerprinting (MLDF; [14,15]) may remain relevant and appear to be sufficiently accurate to assess and monitor population variability and genetic divergence based on polymorphisms of molecular markers. MLDF is a method used to create genetic “fingerprints” grounded in the analysis of DNA polymorphism in the form of restriction fragment length polymorphism (RFLP) markers (e.g., [16,17,18,19,20]). When performing fingerprinting analysis, an oligonucleotide probe of a repetitive (microsatellite) sequence, e.g., (GTG)5, is used, which, in the course of molecular hybridization with genomic DNA, complementarily binds to minisatellite loci, the size of which varies among individuals [21,22]. Using the method of genomic fingerprinting, it is possible to assess the genetic variability and structure of small gene pool populations, as well as to study the origin of some breeds and genetically identify populations. The limitations of this method include the fact that it can only identify anonymous loci and cannot provide as much information as the modern single-nucleotide polymorphism (SNP) genotyping (SNPg) method. However, data obtained in the past using RFLP markers could be used to control population variability in dynamics by comparing the results of previous studies with new SNP-inferred information, particularly if samples from older generations are unavailable for SNPg.

We have at our disposal numerous breeds and populations of divergently selected chickens maintained in the bioresource collection of the Russian Research Institute of Farm Animal Genetics and Breeding (RRIFAGB) and representing a considerable sample of global chicken breed diversity. Among them, there are such established native breeds as Orloff [23], Pavlov [24,25], Yurlov Crower (YC; [26,27,28]), Poltava Clay (PC; [29,30,31,32]), and Russian White (RW; [33,34,35,36,37]). As a consequence of previous phenotypic, genetic, and phylogenetic studies, we obtained information on the genetic diversity of the bioresource collection breeds [22,38,39,40,41,42] as estimated using the MLDF technique, a (GTG)5 probe, and DNA samples collected from some breeds as long as 10 or even 15 years ago. By 2009, our team had assessed genetic diversity in most of these breeds using this method and pairwise comparison of populations to estimate similarity coefficients within and between populations, genetic distances, and heterozygosity. However, due to the incompleteness of the obtained information (not all pairwise combinations of populations were available) and the lack of a complete phylogenetic analysis, it has become necessary to revise the MLDF data and compare them with recent SNPg results for the same breeds [43,44,45,46]. Tyshchenko et al. [47], preliminarily and on a limited scale, compared the use of MLDF method and SNP technology to evaluate changes in the genetic variability of White Cornish and RW breeds over time. Both techniques for assessing genetic diversity in two chicken populations seemed to yield comparable results, although further large-scale tests are required using a much broader sampling of breeds.

In this regard, the aim of this work was to conduct a comparative analysis of the genomic divergence of breeds obtained by two genome-wide assessment methods, namely a general comparison of MLDF data and the results of analysis conducted using SNP chips for most chicken breeds included in the RRIFAGB bioresource collection. This approach enabled us to track changes in breed diversity dynamics. The corresponding phylogenetic trees were plotted for the investigated breeds based on the employed molecular RFLP and SNP markers, and the trees were comparatively evaluated. Several other genetic and genomic variation characteristics were calculated and discussed for the same breeds that were included in these phylogenetic trees.

## 2. Materials and Methods

### 2.1. Animals and DNA Isolation

The subjects of our research were DNA samples obtained from chickens kept in the RRIFAGB collection bioresource farm “Genetic Collection of Rare and Endangered Breeds of Chickens” (Pushkin, St. Petersburg, Russia). Samples for genomic fingerprinting were collected in 2007 from 195 individuals of the following 18 divergently selected breeds/populations (Table 1): Rhode Island Red (RIR), RW, Cochin Blue (CBl), Faverolles Salmon (FS), Moscow Game (MG), New Hampshire (NH), Sussex Light (SL), Uzbek Game (UG), Orloff Mille Fleur (OMF), YC, Pushkin (Pu), Tsarskoye Selo (Ts), Leningrad Golden-and-gray (LGG), Leningrad Mille Fleur (LMF), Zagorsk Salmon (ZS), Pervomai (Pm), Australorp Black Speckled (ABS), and Brahma Light (BL). Samples for genome-wide SNPg were more recently (in 2017) produced from 356 individuals of the same 18 populations (Table 2).

Supplementary Table S1 summarizes population details of the 18 breeds in 2007 and 2017, including population size (total number, i.e., sum of hens and roosters, of 45 to 594), effective population size (25.4 to 299.2), and inbreeding coefficient rate per generation (0.2 to 2.0%). The populations were mainly kept in aviaries, with 45 hens and 5 roosters in small aviaries or 115 hens and 15 roosters in large aviaries. In general, the sex ratio was maintained at 8 hens per rooster. The main mating method was panmixia limited by the selection rate of males at the level of 10–20%. The selection rate of females was not lower than 85%, i.e., only weak and atypical individuals were culled. The remaining shortcomings of hens were corrected by picking the best roosters for them. Groups within a breed were formed if the total population size exceeded 130 heads. Accordingly, the groups were kept in large aviaries of 130 animals (115 hens and 15 roosters) and in small aviaries of 50 animals (45 hens and 5 roosters). The selection of roosters for hens depended on the breeding goal (i.e., homogeneous, heterogeneous, or directionally heterogeneous breeding). When a small number of birds was available in a breed, they were kept in individual cages (i.e., one bird per cage), and individual artificial insemination was used.

Blood samples taken from the brachial wing vein of the studied chicken breeds were used to isolate genomic DNA. Blood samples were collected by qualified laboratory personnel in accordance with the RRIFAGB ethical guidelines to minimize stress or any other disturbance to birds. DNA extraction was performed using the phenol-chloroform method. The quantity and quality of DNA was evaluated using a NanoDrop 2000 spectrophotometer.

### 2.2. Genotyping and Assessment of Genetic Diversity Using MLDF

**Figure 1:**
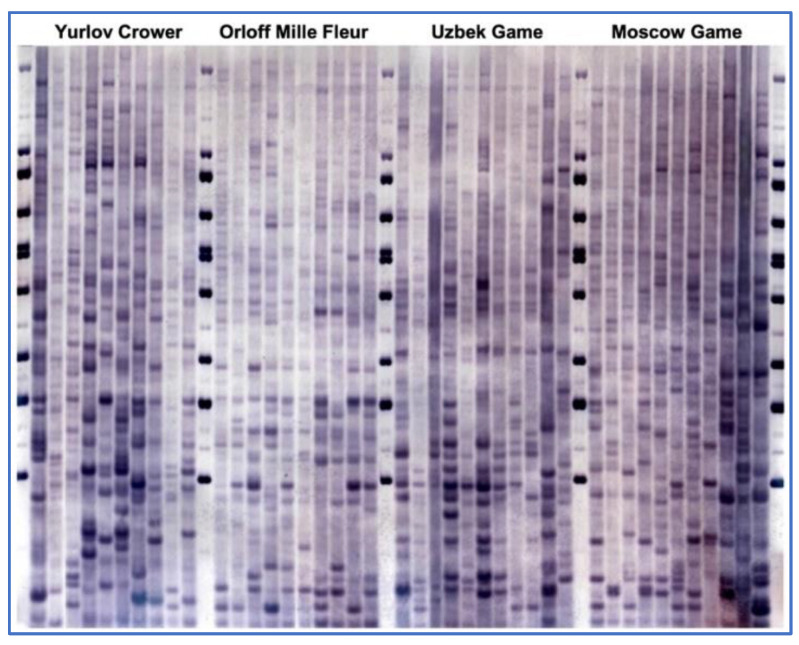
Exemplary multilocus DNA fingerprinting filter generated for within- and between-breed biodiversity evaluation using anonymous RFLP markers in four chicken breeds. DNA fragments resulted from restriction with *Hae*III (or *Bsu*RI), followed by probing with digoxigenin-labelled oligonucleotide (GTG)5. Lanes 1, 12, 23, 35, and 47: DNA ladder (see details in the Section 2.2).

The procedure for genetic analysis by MLDF involved a series of steps as described elsewhere (e.g., [20,48]). Briefly, following the enzymatic digestion of DNA with restriction endonuclease *Hae*III (or its isoschizomer, *Bsu*RI), electrophoresis of DNA fragments was performed using a Sub-Cell Model 192 horizontal high-throughput electrophoresis system (Bio-Rad Laboratories, Inc., Hercules, CA, USA) for 36 h at 60 V in 0.8% agarose gel with a length of 25 cm. These electrophoresis conditions were unified for all experiments, comparing four breeds on each gel (Figure 1). The size-separated fragments were transferred onto a nylon filter, and DNA was fixed on the filter at 80 °C for 1–2 h. Prehybridization was conducted in a special buffer, followed by molecular hybridization in the same buffer containing a deoxygenin-labeled oligonucleotide probe, (GTG)5, at a concentration of 5 pM/mL. Probe binding sites with immobilized DNA fragments were detected via color reaction with dyes. As a DNA ladder, we used lambda phage DNA restriction fragments produced by cleavage with *Hin*dIII and *Bst*EII restriction endonucleases, followed by mixing of the obtained fragments. For improved size control, not two, but up to seven lanes with this DNA marker per gel were used (Figure 1), allowing for a more accurate calculation of the molecular sizes of RFLP bands. The size of RFLP fragments considered in the analysis ranged from 400 to 23,130 bp.

The number and distribution of bands (DNA fragments) on the filter were specific to each individual. Pairwise comparison of all profiles for two different breeds was carried out, and the data were entered into a table for use in calculations. Information on individual filters was combined for the subsequent compilation of a matrix of interbreed distances and the construction of a phylogenetic tree for 18 breeds.

The results of the MLDF analysis were processed using the GELSTATS™ program [49]. Parameters such as *F*_ST_ values (used as genetic distances between breeds and based on band sharing, i.e., the proportion of common DNA fragments within and between breeds [15]) and the mean observed heterozygosity (${\bar{H}}_{O}$) were estimated [50].

The phylogenetic tree was built using the T-REX web server [51] using the algorithm of tree inference from incomplete matrices and the MW * tree reconstruction method [52].

### 2.3. Genotyping and Genetic Diversity Assessment Using SNP Chips

The genomic DNA samples were genotyped using chicken 60K SNP BeadChip^®^ DNA chips produced by Illumina for the GWMAS Consortium [53]. All obtained genotypes were filtered by genotyping quality using the PLINK 1.9 program [54]. Genotypes were with quality exceeding 95% were selected for analysis. The following SNP filtering parameters were applied: --maf, 0.05; --geno, 0.02; and --hwa, 0.0001. After filtering, further analyses were performed using 38,711 SNPs located on autosomes GGA1 to GGA28; SNPs located on sex chromosomes were excluded to avoid their influence on subsequent analyses.

Pairwise *F*_ST_ genetic distances were calculated using the EIGENSOFT program [55] based on SNP profiles obtained from whole-genome screening on Illumina Chicken 60K SNP BeadChip^®^ chips (Illumina, San Diego, CA, USA). Observed (*H_O_*) and expected heterozygosity (*H_E_*), unbiased expected heterozygosity (*_U_H_E_*), allelic diversity (*A_R_*), and coefficient of inbreeding (*F*_IS_) were calculated in the diveRsity R package [56]. Multidimensional scaling (MDS) analysis was performed, the results of which were visualized in the R environment using the ggplot2 library [57].

A phylogenetic tree for 18 breeds was built using the Neighbor-Net algorithm implemented in SplitsTree v. 4.14.6 [58]. This tree was visualized using iTOL v. 5 [59]. Cluster analysis was performed to determine the structure of populations using the Admixture 1.3 program [60]. The results of the admixture analysis were visualized using the POPHELPER package [61].

## 3. Results

### 3.1. MLDF-Based Genetic Diversity

As shown by the results of the genetic diversity study using MLDF, more than 100 DNA fragments per comparison (gel) were detected in the patterns of hybridization of the oligonucleotide probe with chicken genomic DNA (Figure 1). The distribution of these fragments differed between individuals, with a varying probability of occurrence of two individuals having an identical distribution pattern of DNA fragments. This indicator was the lowest in Pu (*p* = 3.20 × 10^−8^) and ZS (*p* = 2.70 × 10^−11^, in two experiments) and the highest in CBl (*p* = 2.34 × 10^−17^), MG (*p* = 1.30 × 10^−17^), and YC (*p* = 1.80 × 10^−18^).

As shown in Table 1, the most recent experimental populations of Ts and LGG had the highest level of mean heterozygosity (${\bar{H}}_{O}$ = 0.82), suggesting increased diversity within these breeds. The highest frequency of occurrence of common DNA fragments was observed in ZS chickens, for which the lowest level of mean heterozygosity was determined (${\bar{H}}_{O}$ = 0.54; Table 1). Populations subject to intensive breeding work (e.g., Pushkin, Tsarskoye Selo, Leningrad Golden-and-gray, Leningrad Mille Fleur, and Australorp Black Speckled) tended to demonstrate lower genetic diversity values (Table 1).

**Figure 2:**
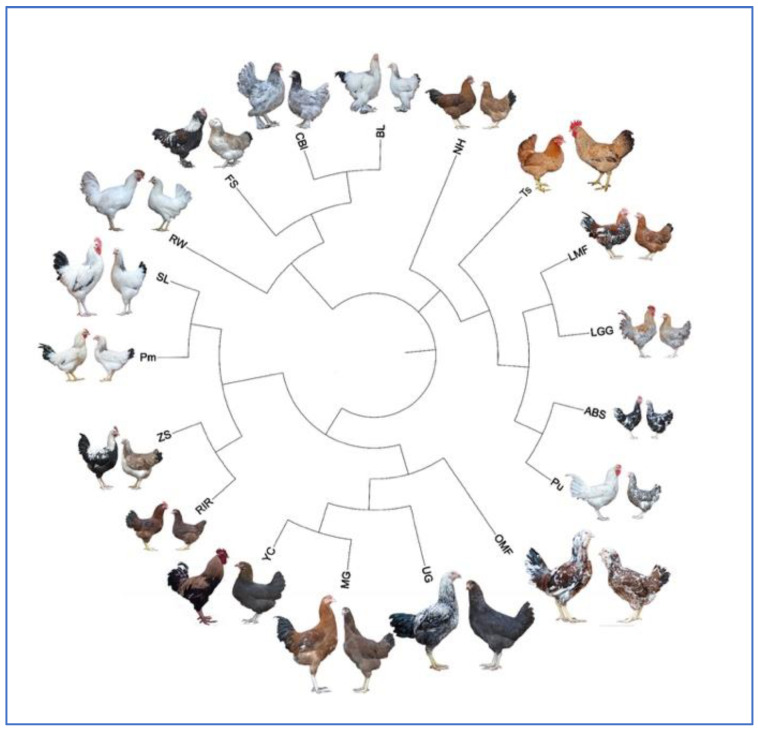
Phylogenetic tree for 18 chicken breeds plotted using pairwise DNA fingerprinting-derived genetic distances (Supplementary Table S2). The corresponding Newick tree format is shown in Supplementary Information S2.

The use of multilocus analysis technology made it possible not only to identify intrapopulation differences but also to calculate genetic distances to build a phylogenetic tree that displays the origin and degree of relationship in 18 divergently selected breeds. The resulting tree (Figure 2) had four main branches and several subclusters, including: (1) Asiatic breeds (BL, CBl), (2) a representative of a European root (egg-type RW), (3) dual-purpose breeds/populations bred at the RRIFAGB (LMF, LGG, ABS, Pu, and Ts), and (4) other dual-purpose breeds (RIR, ZS, Pm, SL) and game breeds (UG, MG, and game-related YC and OMF). For instance, based on the genetic distance between the ABS and Pu populations, these breeds were close to each other, which consistent with the history of breeding, as ABS was one of the breeds that used to create Pu.

In addition, as a result of a complementary analysis using MLDF (Supplementary Information S1), the genetic homogeneity of the RW population from the RRIFAGB farm was revealed, which can be explained by the long-term (over tens of generations) intensive selection for certain traits in this population.

### 3.2. Genetic Diversity Based on SNP Genotypes

The results of the assessment of genetic diversity using DNA chips are shown in Table 2. The lowest *H_O_* value was found in the BL population (0.273 ± 0.001) and the highest in the UG population (0.373 ± 0.002). The *H_E_* and *_U_H_E_* values were also the lowest in BL (0.253 ± 0.001 and 0.261 ± 0.001, respectively), whereas the highest values were in MG (0.354 ± 0.001 and 0.363 ± 0.001, respectively). The lowest *A_R_* value was detected in BL (1.716 ± 0.001), confirming the lower *H_O_* and *H_E_* values in this breed, and the highest *A_R_* value was determined in MG (1.944 ± 0.001). According to the *F*_IS_ coefficient, almost none of the populations manifested inbreeding.

**Figure 3:**
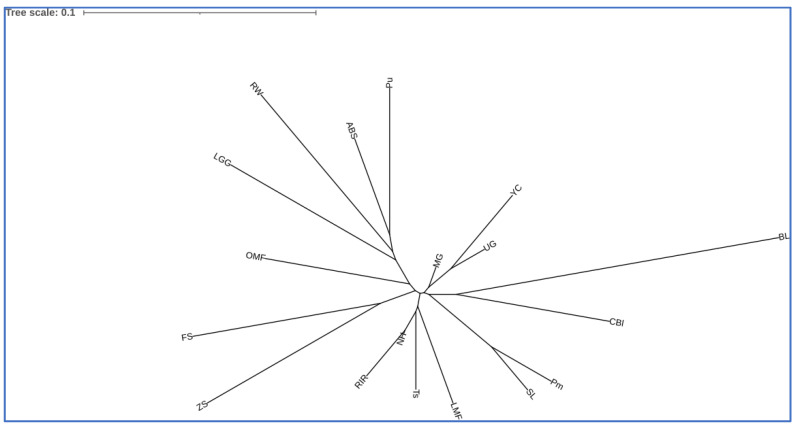
Phylogenetic tree for 18 chicken breeds based on SNP genotypes and pairwise *F*_ST_ genetic distances (Supplementary Table S3) plotted using the Neighbor-Net method (in the SplitsTree 4 program) and the iTOL v. 5 web service [59]. The corresponding Newick tree format is shown in Supplementary Information S2.

**Figure 4:**
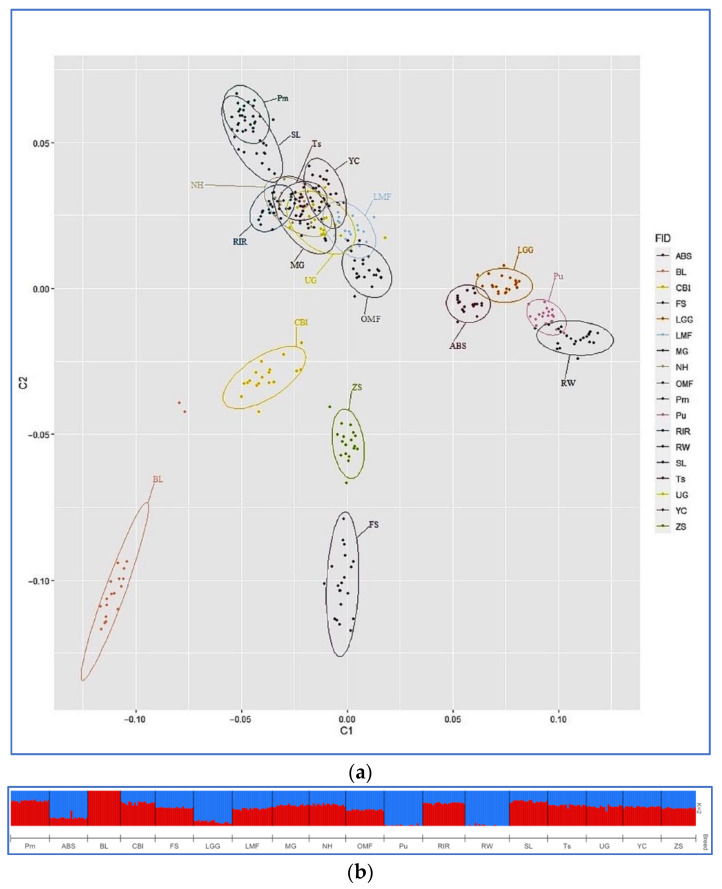
Visualization of the MDS and ADMIXTURE hierarchical clustering based on genotyping information (356 individuals from 18 breeds genotyped for 38,711 SNPs). (**a**) Results of the MDS analysis. Individuals are plotted on the two coordinate axes, C1 and C2, according to their coordinates. Ellipses describe how each breed is dispersed around its center of gravity. (**b**) ADMIXTURE hierarchical clustering analysis for K = 2 predefined clusters (ancestral populations). The proportions of each cluster (*y*-axis) that were defined to be indicative of European (egg-type) and Asiatic (meat-type) ancestries (according to [62]) are plotted in blue and red, respectively, for each individual. Breeds: Pervomai (Pm), Australorp Black Speckled (ABS), Brahma Light (BL), Cochin Blue (CBl), Faverolles Salmon (FS), Leningrad Golden-and-gray (LGG), Leningrad Mille Fleur (LMF), Moscow Game (MG), New Hampshire (NH), Orloff Mille Fleur (OMF), Pushkin (Pu), Rhode Island Red (RIR), Russian White (RW), Sussex Light (SL), Tsarskoye Selo (Ts), Uzbek Game (UG), Yurlov Crower (YC), and Zagorsk Salmon (ZS).

Pairwise *F*_ST_ genetic distances (Supplementary Table S3) ranged from 0.025 (between MG and NH) to 0.278 (between RW and BL). Visualization of pairwise *F*_ST_ genetic distances (Figure 3) showed genetic relatedness between MG, UG, and YC breeds, as well as between SL and Pm and within a group of Pu, ABS, RW, and LGG chickens. Furthermore, interbreed kinship was found between the breed pairs ZS–FS, CBl–BL, and NH–RIR. Similar patterns of breed similarity were observed on the MDS plot of the distribution of individuals based on SNP genotypes (Figure 4a).

### 3.3. Admixture Analysis

**Figure 5:**
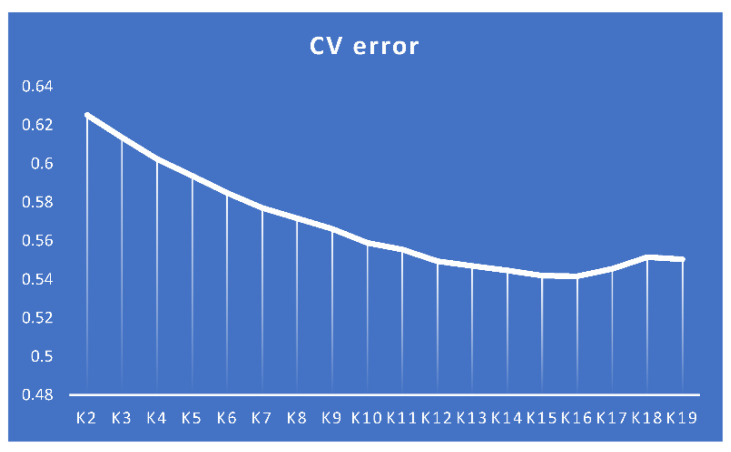
Cross-validation (CV) error in the Admixture analysis based on SNP genotypes in 18 chicken breeds. The optimal number of ancestral populations was K = 16.

**Figure 6:**
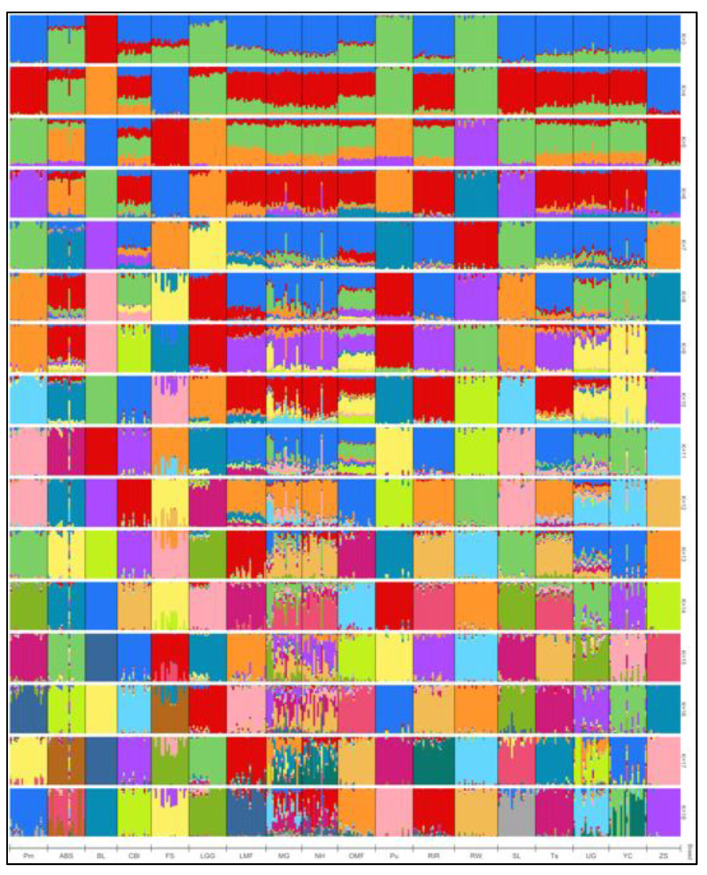
Admixture analysis of the cluster structure conducted for 18 chicken breeds based on genome-wide SNP analysis.

The calculation of the cross-validation error (CV) in the Admixture cluster analysis showed that the most likely number of clusters (ancestral populations) in our breed sample was 16 (Figure 5), which is somewhat out of line with the number of breeds we examined (18). Admixture analysis at K = 2 (Figure 4b) separated BL, Pu, and RW from other breeds. At K = 3, Pm was isolated, and at K = 4, FS and ZS were singled out (Figure 6). Analysis at higher K values showed that MG and NH carry genetic components of different origin. FS had a high percentage of the ZS breed genotypes. Additionally, some individuals of the UG and YC breeds had genetic components of different origins.

### 3.4. Comparative Assessment of Variability Determined by MLDF and SNPg Analysis

**Figure 7:**
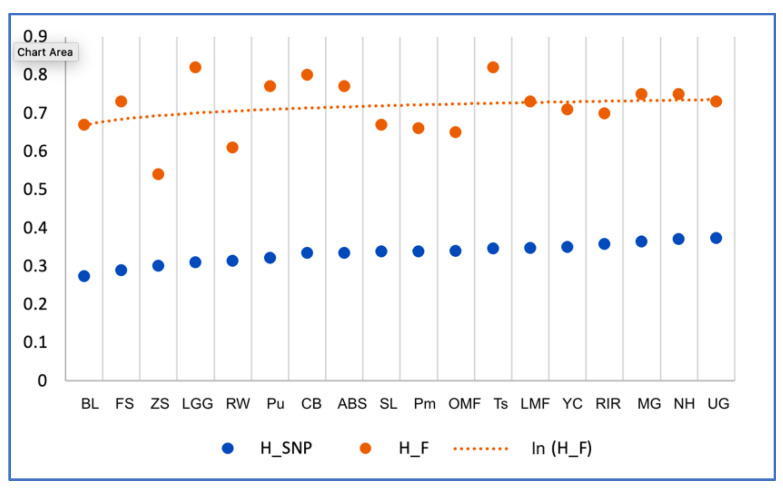
Heterozygosity of 18 populations assessed by various methods: H_SNP, *H_O_* values with SNP genotyping; H_F, ${\bar{H}}_{O}$ values with multilocus DNA fingerprinting; ln (H_F), logarithmic trend y (x)=a×ln (x)+b for the ${\bar{H}}_{O}\;$ curve.

Figure 7 shows a comparison of the indicators of population heterozygosity generated by the two methods for 18 breeds. The analysis showed certain discrepancies in the assessment of variability. Given the estimated values of MLDF-derived heterozygosity (ln-inferred trend line that is largely similar to the SNP curve in Figure 7), the actual values were greater for BL, FS, LGG, Pu, CBl, ABS, and Ts. On the other hand, heterozygosity indicators for ZS, RW, SL, Pm, and OMF showed lower values. This comparison suggested that heterozygosity assessment by MLDF may not be as accurate as that using SNPg. However, it appears that over approximately a decade of breeding the small, closed-gene-pool populations, heterozygosity tended to decrease.

## 4. Discussion

In this work, using the multilocus probe (GTG)5, we performed the fingerprint typing of 18 chicken breeds kept in small, closed gene pool flocks. The use of oligonucleotide (microsatellite) probes, including the (GTG)5 probe employed in this study and others, has been widely established in MLDF investigations. For example, Haberfeld et al. [21] explored the following such probes: a clone R18.1 from the bovine genome containing poly (GT) sequences, as well as oligonucleotide probes (GTG)5 and (GT)12. Hybridization of the R18.1 probe with *Hin*fI restricted chicken DNA revealed a highly polymorphic pattern of DNA fingerprinting. In 20 unrelated broilers, an average of 27.8 bands per individual was detected in the range of 3 to 23 kb. Additionally, according to Epplen et al. [22], the best probes to obtain fingerprinting patterns in chickens were (GGAT)4, (GTG)5, and (TCC)5. We suggest that MLDF is the most suitable method when the tendency of similarity of genotypes in one breed is clearly visible when breeding very small groups of animals. For example, in a very small population of BL chickens, we analyzed even in the 1990s, we observed a very low heterozygosity and, most likely, a high degree of inbreeding. The same breed showed a higher heterozygosity estimate in more recent MLDF studies (2009) and lower heterozygosity in the recent SNP survey presented here. Previously, we were only able to partially describe the MLDF results in our studies [39,63,64], whereas additional analysis in the present study (using the algorithm of tree inference from incomplete matrices; [52]) enabled us to fill in all the gaps in the assessment of interbreeding variability and phylogeny.

A comparative evaluation of ${\bar{H}}_{O}$ data obtained using MLDF and variability determined by SNP analysis showed a decline in heterozygosity in the populations of BL, FS, LGG, Pu, CBl, ABS, Ts, and other breeds, possibly due to a decrease in the population size of BL, FS, ABS, Pu, and CBl breeds observed from 2007 to 2017. An intensive selection in the experimental LGG and Ts populations appears to have led to a decrease in their heterozygosity. According to the MLDF data, ${\bar{H}}_{O}$ in ZS, RW, SL, Pm, and OMF was slightly lower, as a result of the effort to restore their genetic diversity, including measures such as individual selection and purchase of breeding eggs from other collections from 2007 to 2017. The importance of monitoring genetic variability for the timely adjustment of breeding priorities in small populations has also been emphasized by other authors [65,66].

As for the MLDF procedure itself, it is well established that it is subject limitations and drawbacks. If it were a universal, comprehensive, highly reliable, straightforward, and highly effective method, it would still be widely used today. However, it has been gradually discontinued since the early 2000s and is rarely revisited. This technique was largely abandoned because it used anonymous loci and was difficult to perform (setting up and conducting one experiment took almost a week), and only few samples could be compared at the same time (as it is desirable to conduct comparisons on the same filter). However, based on our many years of experience (e.g., [47]) and this comparative study, we suggest that MLDF could be an adequate method to compare related breeds or the level of population variability over time. Furthermore, the efficacy of assessment of inbreeding by MLDF is doubtful, and it is disputable that MLDF is an appropriate method that can reliably detect the degree of inbreeding, as we previously showed in another comparative study [47] and as observed in other investigations [67,68,69,70].

Nevertheless, in this work, we found a suitable way to analyze previous fingerprints and build the corresponding phylogenetic tree using the tree inference from incomplete matrices for 18 divergently selected breeds surveyed in the 2000s. The resulting tree topology (Figure 2) was largely congruent to that for the trees that were constructed based on the SNPg data of the same 18 populations (Figure 3). This topology also echoed the phylogeny that was produced by Larkina et al. [71] for the same breeds. Therefore, in phylogenetic terms, it seems reasonable to suggest that an obsolete MLDF method was adequate to compare variability between breeds and populations in dynamics. Therefore, the phylogenetic results described here enabled the evaluation of the effectiveness of analysis based on DNA fingerprints and SNP markers in a comparative aspect. To the best of our knowledge, such an analysis of the domestic gene pool (more precisely, an extensive sample of the world gene pool) has not been performed to date. In the previous study [71], we only slightly attempted to approach such a comparative analysis but using different datasets, i.e., in terms of multiple phenotypic traits and five SNPs at a single locus.

The two phylogenetic trees generated with RFLP markers (Figure 2) and SNPs (Figure 3) had a number of significant overlaps. There were also differences that could occur for two main reasons. First, SNP markers provide more extensive genome coverage and a more accurate display of genomic variability. Second, there were several gaps in the RFLP data, which (although they were filled virtually using a computer algorithm) could become an additional source of errors in the reconstruction of phylogenetic relationships.

The presented analysis of two approaches to assess genetic diversity provides a deeper understanding of the features of the genomic architecture of chicken breeds and populations. In addition, we compared the degree of variability within populations using two methods and correlated the results with historical breed data. The two approaches used in our study to assess genetic relatedness between chicken populations revealed differences between Asiatic breeds (e.g., BL and CBl) and genetically rooted light-layer breeds, such as RW and Pu. Similar results were reported in a study by Malomane et al. [72].

The optimal probable number of admixture-inferred clusters (i.e., 16 ancestral populations; Figure 5) did not match the actual number of breeds we explored (18), possibly due to problems in the breeding process of different breeds related to the occurrence of random, unplanned crossings between populations similar in phenotype. At K = 2 (Figure 4b), the Asiatic meat BL breed was clearly distinguished, as well as two breeds of RW and Pu descended from White Leghorns that represent the egg-layer (Mediterranean) ancestral type. The rest of the breeds had varying proportions of genomic “mixing” of these two ancestral types. Thus, at K = 2, we have a subdivision of all the studied breeds in strict accordance with the two primary evolutionary branches of chicken breed formation as was postulated by Moiseyeva et al. [62]. At K = 3 (Figure 6), a third ancestral type was added that most likely conforms to the dual-purpose breeds proposed by Larkina et al. [71] as another major evolutionary breed-formation branch. Accordingly, the following four groups of populations can be distinguished: (1) meat-type BL, as well as CBl and FS with meat-type “impurities”; (2) egg-type RW and Pu, with adjoining experimental populations of LGG and ABS; (3) typical dual-purpose breeds SL and Pm (which, due to the common Columbian plumage coloration, could also interbreed uncontrollably); and (4) all other breeds showing a varied degree of mixed ancestry. Separation of FS and ZS at K = 4 that were partially unseparated at K = 8 evidenced an admixture of ZS genotypes in the FS population. Analysis at the maximum K values showed that MG and NH carry numerous genetic components of differing origins, possibly as a result of significant random mixing of the two breeds with each other and with other breeds. The discovery of such introgression of breeds is common in poultry breeding; many researchers studying the genetic diversity of chicken breeds and populations have reached similar conclusions (e.g., [2,3,73]).

Additionally, some individuals of the UG and YC breeds had genetic components of different origins, indicating the need for further study of these breeds. The first of these is a game breed, and the second has game chicken roots in its origin. The game type also stands out as an independent evolutionary branch of domestic chicken breeding according to the concept proposed by Moiseyeva et al. [62]; therefore, in general, we can suggest that in the present study, there was a formation of a separate cluster that consisted of game and related chicken breeds. This suggestion is also supported by our phylogenetic/clustering models (Figure 2, Figure 3 and Figure 4), which generally showed genetic relationships between these breeds, including MG, UG, YC, and OMF. A close relationship between the following pairs (groups) of breeds can be observed in the admixture bar plots, phylogenetic trees, and MDS plot: (1) SL and Pm, which were partly interbred; (2) a group of layers and related breeds, including RW, Pu, ABS, and LGG; (3) a branch of Asiatic breeds CBl and BL; (4) ZS and FS, which (as previously noted) also merged into a separate subcluster; and (5) related populations of NH and RIR, with NH exhibiting significant interbreeding in its demographic history.

Collectively, when comparing phylogenies from MLDF and genome-wide SNPg, a clear concept of the distribution within this large global sample of chicken breeds can be tracked, which conforms to known models of their evolutionary subdivision [62,71]. On the other hand, there were also discrepancies: for example, in the tree plotted on the basis of the SNPg results (Figure 3), BL was in the same subcluster with CBl, whereas FS was clustered with ZS; however, in the MLDF-inferred tree BL was placed on the same branch with CBl and FS (Figure 2). Furthermore, as shown in Figure 2, RW was located largely apart from the rest of the breeds, whereas in Figure 3 this breed was situated next to Pu, ABS, and LGG, probably due to the common roots of origin and the focus of selection in these populations on egg production, which was better displayed using a more advanced SNPg method.

## 5. Conclusions

In the present study, we attempted to assess the biodiversity of a large number of chicken breeds from the RRIFAGB bioresource collection. In genetic and phylogenetic studies of 18 divergently selected breeds, we not only assessed the general phylogeny of breeds but also considered how two types of markers were able to reflect these phylogenetic relationships. On the basis of MLDF data, we obtained a matrix of interbreed distances, calculated population genetic indicators characterizing the genetic variability of breeds, and built phylogenetic trees and cluster models. Our analysis results show that the MLDF method remains a fairly accurate method for retrospective analysis of changes in the genetic divergence of populations over time, although the method itself is already very outdated. Although there are examples of comparisons of multilocus DNA fingerprints and microsatellites to determine genetic distances in chicken [19], such a comparison of RFLP and SNP markers within the same work, to the best of our knowledge, has not been carried out to day (except for our limited preliminary survey of two breeds; [47]). Therefore, this comparative aspect was somewhat novel in phylogeny studies of chickens. The estimates of genetic diversity obtained as a result of the analysis of two approaches provides a deeper understanding of the features of the genomic architecture of chicken breeds and populations. In addition, we emphasize the particular importance of constantly monitoring the variability of small-gene-pool populations and comparing these results with data on the origin and demographic history of breeds in order to develop the most effective germplasm innovation strategy.

## Figures and Tables

**Table 1:** Mean heterozygosity (${\bar{H}}_{O}$) and other genetic diversity indicators computed using the GELSTATS™ program in 18 chicken breeds analysis of fingerprinting patterns.

Breed	Abbreviature	*n* ^1^	${\bar{H}}_{O}$	Mean No. of Loci per Individual	Mean No. of Alleles Per Locus	Proportion of Polymorphic Loci
Rhode Island Red	RIR	11	0.70	19.49	5.44	0.94
Russian White	RW	11	0.61	18.38	4.79	0.95
Cochin Blue	CBl	11	0.80	16.44	6.99	1.00
Faverolles Salmon	FS	11	0.73	16.62	6.26	1.00
Moscow Game	MG	11	0.75	20.81	5.62	1.00
New Hampshire	NH	11	0.75	14.15	7.62	1.00
Sussex Light	SL	11	0.67	16.50	5.69	0.88
Uzbek Game	UG	11	0.73	19.23	5.72	1.00
Orloff Mille Fleur	OMF	10	0.65	18.21	5.11	0.95
Yurlov Crower	YC	10	0.71	20.92	5.62	1.00
Pushkin	Pu	11	0.77	11.35	5.99	1.00
Tsarskoye Selo	Ts	11	0.82	9.28	7.97	1.00
Leningrad Golden-and-gray	LGG	11	0.82	9.56	8.01	1.00
Leningrad Mille Fleur	LMF	10	0.73	10.55	6.54	1.00
Zagorsk Salmon	ZS	11	0.54	20.49	4.72	0.77
Pervomai	Pm	11	0.66	17.15	5.30	0.94
Australorp Black Speckled	ABS	11	0.77	12.39	6.46	0.92
Brahma Light	BL	11	0.67	17.11	6.08	0.88

^1^ *n*, number of genotyped birds.

**Table 2:** Genetic diversity scores (M ± SE) based on SNP genotypes in the 18 studied chicken populations ^1^.

Breed	*n*	*H_O_*	*H_E_*	*_U_H_E_*	*A_R_*	*F*_IS_
RIR	22	0.358 ± 0.001	0.338 ± 0.001	0.346 ± 0.001	1.918 ± 0.001	−0.054 ± 0.001
RW	23	0.314 ± 0.001	0.294 ± 0.001	0.301 ± 0.002	1.848 ± 0.001	−0.063 ± 0.001
CBl	18	0.335 ± 0.001	0.315 ± 0.001	0.324 ± 0.001	1.867 ± 0.001	−0.059 ± 0.002
FS	20	0.289 ± 0.001	0.295 ± 0.001	0.302 ± 0.001	1.831 ± 0.002	0.012 ± 0.001
MG	19	0.364 ± 0.002	0.354 ± 0.001	0.363 ± 0.002	1.944 ± 0.001	−0.028 ± 0.001
NH	19	0.371 ± 0.001	0.351 ± 0.002	0.361 ± 0.001	1.939 ± 0.001	−0.053 ± 0.001
SL	20	0.338 ± 0.001	0.321 ± 0.001	0.329 ± 0.001	1.882 ± 0.001	−0.048 ± 0.001
UG	19	0.373 ± 0.002	0.343 ± 0.001	0.353 ± 0.001	1.922 ± 0.001	−0.078 ± 0.001
OMF	20	0.340 ± 0.001	0.322 ± 0.001	0.330 ± 0.002	1.891 ± 0.001	−0.052 ± 0.001
YC	20	0.350 ± 0.001	0.326 ± 0.001	0.334 ± 0.001	1.889 ± 0.002	−0.068 ± 0.002
Pu	20	0.322 ± 0.001	0.303 ± 0.001	0.311 ± 0.001	1.836 ± 0.001	−0.056 ± 0.002
Ts	20	0.346 ± 0.002	0.333 ± 0.001	0.342 ± 0.001	1.905 ± 0.001	−0.038 ± 0.001
LGG	20	0.310 ± 0.001	0.295 ± 0.001	0.303 ± 0.001	1.827 ± 0.001	−0.046 ± 0.001
LMF	21	0.348 ± 0.001	0.329 ± 0.001	0.337 ± 0.001	1.898 ± 0.001	−0.056 ± 0.001
ZS	18	0.301 ± 0.001	0.286 ± 0.002	0.295 ± 0.001	1.816 ± 0.002	−0.047 ± 0.001
Pm	20	0.338 ± 0.001	0.317 ± 0.001	0.326 ± 0.001	1.875 ± 0.001	−0.061 ± 0.001
ABS	20	0.335 ± 0.001	0.321 ± 0.001	0.329 ± 0.001	1.887 ± 0.001	−0.036 ± 0.001
BL	17	0.273 ± 0.001	0.253 ± 0.001	0.261 ± 0.001	1.716 ± 0.001	−0.072 ± 0.001

^1^ M, mean value; SE, standard error; *n*, number of genotyped birds; *H_O_*, observed heterozygosity; *H_E_*, expected heterozygosity; *_U_H_E_*, unbiased expected heterozygosity; *A_R_*, rarefied allelic richness; *F*_IS_, inbreeding coefficient.

## Data Availability

The MLDF genotyping datasets are available from the figshare repository: https://doi.org/10.6084/m9.figshare.20736760 (accessed on 13 October 2022). The proprietary SNP genotyping data produced and analyzed in this study were generated using the 60K chicken SNP chip produced by Illumina Inc. for the GWMAS Consortium represented by Cobb-Vantress Inc. and Hendrix Genetics B.V. As such, the datasets generated using this chip are confidential and protected as intellectual property or as trade secrets. As a consequence, the SNP genotyping information used in this study was not made public but is kept in a secure database at the RRIFAGB. However, the data can be provided upon reasonable request and can be shared with third parties upon approval from the GWMAS Consortium. The authors affirm that all other data necessary to confirm conclusions reported in this article are present within the article, figures, and tables.

## Supplementary Materials

The following supporting information can be downloaded at: https://www.mdpi.com/article/10.3390/genes13101876/s1, Supplementary Information S1: Evaluation of the genetic variation in Russian White chickens using DNA fingerprinting; Supplementary Information S2: Newick format for representation of phylogenetic trees; Table S1: Population characteristics of the chicken germplasm breeds in 2007 and 2017 [74,75]; Table S2: Pairwise genetic *F*_ST_ distances between the 18 studied chicken breeds based on multilocus DNA fingerprinting; Table S3: Pairwise genetic *F*_ST_ distances between the 18 studied chicken breeds based on SNP genotypes.
