# Supplementary material for: Comparative Analysis of Molecular RFLP and SNP Markers in Assessing and Understanding the Genetic Diversity of Various Chicken Breeds
Source: Genes (Basel). 2022 Oct 16;13(10):1876. doi: 10.3390/genes13101876 (PMC9601448; doi:10.3390/genes13101876)
Supplement: Supplementary file 1 [file genes-13-01876-s001.zip › Suppl Information S1.pdf]

Supplementary Information S1: Evaluation of the genetic variation in Russian White chickens using DNA fingerprinting

Goal setting. Since a chicken population of the Russian White (RW) breed kept at the Genofond farm of the Russian Research Institute of Farm Animal Genetics and Breeding (RRIFAGB), Pushkin, St. Petersburg, was bred from 2002 to 2012 by panmixia and the previous linear structure of the breed, with subdivision into two Lines 10 and 16, was lost, it was of great interest to compare the specific features of the RW breed genome in past years with those in the existing population.

Samples. For this purpose, samples of 2001 collected from the RW population with a linear structure, i.e., from the RW Lines 10 and 16 (further considered here as subpopulations, too), were preliminarily analyzed. For a comparative assessment of the differences between the RW subpopulations, the archived DNA samples of the same breed were additionally used that were isolated from blood samples provided in 2001 by the Genofond farm of the All-Russian Poultry Research and Technological Institute (ARPRTI), Sergiev Posad, Moscow Region.

DNA fingerprinting. This work was carried out using a deoxygenin-labeled oligonucleotide probe (GTG)5, which made it possible to identify hypervariable regions of the breed's genome. Calling the distribution of DNA fragments was done using the RFLPscan™ computer program (Stratagene Cloning Systems, La Jolla, CA, USA) that was able to detect and compare the position of DNA fragments on a nylon filter (Fig. S1-1). The threshold level was set within 1% (i.e., fragments differing by more than 1% in their length were considered different).

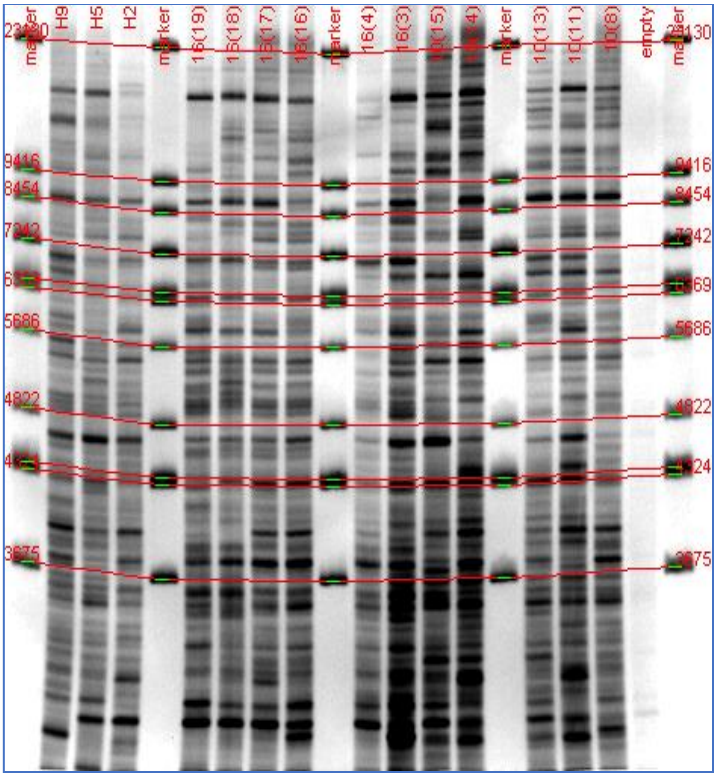

Figure S1-1. DNA fingerprints of RW chickens. Samples: crossbreds between Lines 10 and 16, lanes 2–4 (individuals H9, H5 and H2); Line 16, lanes 6–9, 11 and 12 (individuals 19, 18, 17, 16, 4 and 3); Line 10, lanes 13, 14 and 16–18 (individuals 15, 14, 13, 11 and 8); and DNA size marker, lanes 1, 5, 10 and 20.

Mathematical analyzes and tests. Using fingerprinting filters from the archived samples, the main population genetic parameters were computed, such as the coefficient of similarity within and between subpopulations, heterozygosity, allele frequencies, F_{ST}, etc. The calculations were based on non-parametric statistics using the method of permutations. Number of permutations in all cases was set at the level of 5,000, while a further increase did not lead to changes in the calculated data. The nonparametric test used by the program had certain advantages over other approaches in the analysis of the distribution of DNA fragments. The use of complex statistics was justified for unknown distributions and for small samples. In addition, this approach was

justified for a different number of individuals in groups and a different number of DNA fragments. At the same time, the permutation method avoided the data dependency problem discussed elsewhere in the literature (Lynch 1990, 1991). Permutation is considered to be a more powerful statistical approach than non-parametric statistics using ranks. Also, permutation tests provide accurate probabilities (Rogstad and Pelikan 1996).

The program computed F_{ST} values based on the fundamental work of Lynch (Lynch 1991), in which it was found that this parameter, showing the level of subdivision of a population into subpopulations, can be calculated using the following formula:

$$F_{ST} = (1 - S_b) / (2 - S_b - S_w),$$

where S_b is a similarity score between groups and is determined by calculating the similarity of DNA fragments in pairwise comparisons of individuals in different groups, and S_w is defined as the average proportion of common DNA fragments in pairwise comparisons in each of the groups, followed by averaging over all groups.

Thus, S_w is the average of all S_{ij} values calculated for all compared pairs of subpopulations i and j:

$$S_{ij} = 1 + S'_{ij} - (S_i + S_j) / 2,$$

where S_i and S_j are the average similarities of individuals in groups i and j.

Additionally, the similarity coefficient BS (band sharing) was determined as the ratio of the doubled number of common bands to the total amount of bands in a pairwise comparison:

$$BS = 2B_{xy} / (B_x + B_y),$$

where BS is the coefficient of similarity (or mean similarity), B_{xy} is the number of identical bands in the compared two animals, and B_x and B_y are the total number of bands in animal x and y, respectively.

The genetic distance between populations/subpopulations was also determined (D):

$$D = (BS_1 + BS_2) / 2 - BS_{1,2}$$

The GELSTATS™ program (Rogstad and Pelikan 1996) also made it possible to calculate the F_{ST} values according to Nei (1973) in the RRIFAGB population.

Results and discussion. The value of the F_{ST} parameter (computed in GELSTATS™ according to Nei 1973) turned out to be 0.105, which also indicates the subdivision of the RRIFAGB population into subgroups (i.e., lines) in accordance with the implemented selection goals.

Interestingly, when comparing two different RW subpopulations, there was a significant genetic distance between Lines 10 and 16 (D = 0.115) calculated using the interline similarity coefficient (BS; Table S1-1) from the above formula, which was sometimes consistent with the differences we observed previously between certain chicken breeds. An even greater remoteness was observed for the compared RRIFAGB and ARPRTI populations. The genetic distance D between the unique Line 16 and the ARPRTI population was 0.175.

Table S1-1. Values of population genetic parameters calculated using the GELSTATS™ program in chicken groups (lines) and between RW lines.[1]

Pairwise comparisons	Populations (subpopulations)	n	No. of bands per lane ($M \pm m$)	Similarity coefficient (BS)	
				Within groups	Between groups
1	RRIFAGB Line 10	5	18.0 ± 7.7	0.52^{a}	0.46^{d}
	RRIFAGB Line 16	5	19.4 ± 4.3	0.63^{b}	
2	ARPRTI group	10	16.4 ± 3.2	0.46^{c}	0.39^{e}
	RRIFAGB Line 10	5	18.0 ± 7.7	0.52	
3	RRIFAGB Line 16	5	19.4 ± 4.3	0.63	0.37^{f}
	ARPRTI group	10	16.4 ± 3.2	0.46	

[1] n, number of genotyped birds; M, mean value; SE, standard error. Significance of intergroup differences: $^{a,\,b}$ $p < 0.001$; $^{a,\,e}$ $p < 0.01$, $^{b,\,c}$ $p < 0.01$; $^{b,\,d}$ $p < 0.001$; $^{b,\,f}$ $p < 0.001$; $^{c,\,f}$ $p < 0.05$.

A lower level of heterozygosity was found in Line 16 ($\bar{H}_O$ = 0.37; Table S1-2), which may indicate a higher genetic homogeneity within this line. For comparison, in the ARPRTI population, the level of heterozygosity was significantly higher ($\bar{H}_O$ = 0.66). In this regard, it would be interesting to compare these conclusions in the future with the data obtained during genotyping of this and other breeds using SNP chip technology, taking into account tens of thousands of available SNP loci. If the assessment of variability by these two methods coincided, it would be possible to trace the dynamics of variability in small populations over 5–15 years without additional research (using the existing research backlog since 2001).

Table S1-2. Average heterozygosity ($\bar{H}_O$) in the RRIFAGB lines and ARPRTI population calculated by the GELSTATS™ program.

Populations (subpopulations)	Number of loci	Number of alleles per locus	$\bar{H}_O$
RRIFAGB Line 10, *n* = 5	12	3.3	0.51[a]
RRIFAGB Line 16, *n* = 5	14	2.5	0.37[b]
ARPRTI group, *n* = 10	9	5.6	0.66[c]

Significance of intergroup differences: [a, c] p < 0,001; [b, c] p < 0,001.

Conclusions. In the present study, we computed the genetic variability of the archived DNA fingerprints in the RW chickens including the ARPRTI population and RRIFAGB Lines 10 and 16. In the future, it would be desirable to compare these findings with data obtained from SNP genotyping using tens of thousands of SNP markers. Such work would be necessary to assess the possibility of extrapolation of the results on the genetic diversity of poultry obtained by the method of multilocus DNA fingerprinting in 1996–2010 to the data of genetic characterization based on SNPs in current small gene pool populations of chickens.

If there were a coincidence in evaluation of variability by these two methods, it would be possible, without additional research, to trace the dynamics of variability in small populations over 5–20 years. Overall, the genome-wide characterization of populations and breeds for a variety of SNP loci will constitute the further basis for developing and implementing genomic selection and effectively utilizing the potential of domestic gene pool in the poultry industry.
