# Supplementary material for: Comparative Analysis of Molecular RFLP and SNP Markers in Assessing and Understanding the Genetic Diversity of Various Chicken Breeds
Source: Genes (Basel). 2022 Oct 16;13(10):1876. doi: 10.3390/genes13101876 (PMC9601448; doi:10.3390/genes13101876)
Supplement: Supplementary file 1 [file genes-13-01876-s001.zip › Suppl Information S2.pdf]

Supplementary Information S2: Newick format for representing phylogenetic trees

Figure 2 tree:
(((OMF:0.0553,(UG:0.0369,(YC:0.0232,MG:0.0267):0.0031):0.0211):0.0000,((SL:0.0581,Pm:0.0370):0.0166,(ZS:0.0553,RIR:0.0497):0.0059):0.0065):0.0126,(NH:0.0464,(Ts:0.0387,((Pu:0.0318,ABS:0.0182):0.0302,(LGG:0.0480,LMF:0.0420):0.0088):0.0037):0.0093):0.0120,(RW:0.1042,(FS:0.0506,(BL:0.0486,CBl:0.0364):0.0044):0.0360):0.0330);

Figure 3 tree:
(((((RIR:0.02572916666666665,NH:0.001270833333333346):0.009378906249999971,Ts:0.033621093750000025):0.002323660714285712,LMF:0.04442633928571428):0.005675781250000001,((((RW:0.0881517857142857,(Pu:0.06308333333333335,ABS:0.043916666666666646):0.007348214285714291):0.003942708333333322,LGG:0.08205729166666667):0.011975260416666662,OMF:0.06302473958333339):0.0037880859375000013,(FS:0.08198076923076919,ZS:0.08601923076923083):0.01602441406249997):0.002359374999999995):0.0017773437499999933,(MG:0.009096875000000011,(UG:0.016676136363633,YC:0.04132386363636367):0.012403124999999994):0.0030917968750000108,((CBl:0.06696875,BL:0.14103125000000002):0.011796874999999957,(SL:0.024312500000000015,Pm:0.029687499999999978):0.035203125000000003):0.0021582031250000017);
