# Supplementary material for: Comparative Analysis of Molecular RFLP and SNP Markers in Assessing and Understanding the Genetic Diversity of Various Chicken Breeds
Source: Genes (Basel). 2022 Oct 16;13(10):1876. doi: 10.3390/genes13101876 (PMC9601448; doi:10.3390/genes13101876)
Supplement: Supplementary file 1 [file genes-13-01876-s001.zip › Suppl Table S1.pdf]

Table S1. Population characteristics[1] of the chicken germplasm breeds in 2007 and 2017.

Breeds	Populations in 2007					Populations in 2017				
	No. of birds per breed			N_e	ΔF, %	No. of birds per breed			N_e	ΔF, %
	♀	♂	Total			♀	♂	Total		
Australorp Black Speckled	214	26	240	92.7	0.5	86	13	99	45.2	1.1
Brahma Light	46	13	59	40.5	1.2	37	8	45	26.3	1.9
Cochin Blue	89	15	104	51.3	1.0	57	10	67	34.0	1.6
Faverolles Salmon	42	9	51	29.6	1.7	67	12	79	40.7	1.2
Leningrad Golden-and-gray	308	38	346	135.3	0.4	130	15	145	53.8	0.9
Leningrad Mille Fleur	173	23	196	81.2	0.6	253	46	299	155.7	0.3
Moscow Game	43	10	53	32.5	1.5	98	17	115	57.9	0.9
New Hampshire	147	17	164	61.0	0.8	85	15	100	51.0	1.0
Orloff Mille Fleur	42	12	54	37.3	1.3	134	23	157	78.5	0.6
Pervomai	157	18	175	64.6	0.8	48	10	58	33.1	1.5
Pushkin	575	86	661	299.2	0.2	264	32	296	114.2	0.4
Rhode Island Red	106	14	120	49.5	1.0	156	50	206	151.5	0.3
Russian White	104	17	121	58.4	0.9	196	40	236	132.9	0.4
Sussex Light	135	15	150	54.0	0.9	68	7	75	25.4	2.0
Tsarskoye Selo	103	19	122	64.2	0.8	252	41	293	141.1	0.4
Uzbek Game	44	11	55	35.2	1.4	115	20	135	68.1	0.7
Yurlov Crower	531	63	594	225.3	0.2	194	34	228	115.7	0.4
Zagorsk Salmon	194	21	215	75.8	0.7	89	10	99	36.0	1.4

[1] N_e, effective population size estimated by the formula $N_e = 4N_mN_f/(N_m + N_f)$, where N_m is the number of males and N_f is the number of females [74]; ΔF, rate of inbreeding increase per generation calculated by the formula by $\Delta F = 1/(2N_e)$, where F is the inbreeding coefficient [75], and multiplied by 100%.

References

74. Kliman, R.; Sheehy, B.; Schultz, J. Genetic drift and effective population size. *Nature Educ.* **2008**, *1*(3):3.
75. Falconer, D.S. *Introduction to Quantitative Genetics*, 3rd ed. Longman: New York, USA, 1989.
